# Geographic and seasonal variation in malaria prevalence on islands in Lake Victoria (western Kenya): results from three cross sectional studies

**DOI:** 10.1186/1475-2875-13-S1-P61

**Published:** 2014-09-22

**Authors:** Zulkarnain Md Idris, Wai Chan Chim, Sheng Deng Chang, Kimura Masatsugu, Teramoto Isao, Omar Ahmediin, Akhwale Willis, Kongere James, Kaneko Akira

**Affiliations:** 1 Department of Microbiology, Tumor and Cell Biology, Karolinska Institutet, Stockholm, Sweden; 2 Department of Parasitology and Medical Entomology, Faculty of Medicine, Universiti Kebangsaan Malaysia, Kuala Lumpur, Malaysia; 3 Research Center of Qinghao, Guangzhou University of Chinese Medicine, Guangzhou, China; 4 Department of Parasitology, Graduate School of Medicine, Osaka City University, Osaka, Japan; 5 Division of Malaria Control, Ministry of Public Health and Sanitation, Nairobi, Kenya

## Background

Kenya launched its second National Malaria Strategy with a notably ambitious vision for a “malaria free Kenya”, but malaria remains a major health problem among communities in Lake Victoria in western Kenya.

## Materials and methods

We conducted three cross-sectional surveys during the dry (January 2012) and two consecutive wet seasons (August 2012 and August 2013) among communities residing in three different settings in Lake Victoria: (1) along the mainland coast (Ungoye: population ~2,000); (2) on a large island (Mfangano: population ~25,000); and (3) on small islands (Takawiri, Kibuogi, and Ngodhe: population ~700 each). The prevalence of malaria infections and G6PD deficiency was analyzed by geography and/or season.

## Results

Overall, parasite rates (PRs) as determined by microscopy (18.9% vs 18.4%), rapid diagnostic test (RDT; 36.9% vs 30.8%) and PCR (31.1% vs 28.5%) were higher in the dry season than in the wet seasons with characteristic age distribution. In both seasons, PRs were highest in the coast, then in the large island and lowest on the small islands. The highest prevalence by RDT during the dry season was observed in Ungoye: 54.4% in age group 0-5 years old, 68.4% in 6-10, 55.3% in 11-15, 13.2% in 16-30 and 11.3% in >30. Species-specific prevalence by PCR was 29.3% for *Plasmodium falciparum*, 8.5% for *P. malariae*, and 2.1% for *P. ovale* in the dry season, and 26.6%, 7.2%, and 3.3%, respectively, in the wet seasons. Prevalence of mixed infections was 8.3% and 7.1% in the dry and wet seasons, respectively. *P. vivax* was not detected by microscopy or PCR in any survey. Significant seasonal fluctuations in PRs were observed among children and young adolescents on islands but not in the coast. Among all settings, G6PD deficiency was found in 12.1% of males and 2.1% of females. No significant correlation between G6PD deficiency rates and PRs was observed in any settings.

## Conclusions

Variation in malaria prevalence reflects the different dynamic of malaria transmission between the islands and the coast of Lake Victoria. Our results provide baseline data for the planned feasibility study of malaria elimination on islands in Lake Victoria.

